# Exploring causal correlations between plasma proteins and peripheral neuropathy: a Mendelian randomization

**DOI:** 10.3389/fneur.2024.1431669

**Published:** 2024-08-29

**Authors:** Man Song, Fang Chen, Xiaocong Li, Lu Chen

**Affiliations:** ^1^Department of Intensive Care Unit, Baoji Hospital of Traditional Chinese Medicine, Baoji, China; ^2^Department of Neurology, General Hospital of Ningxia Medical University, Yinchuan, China

**Keywords:** peripheral neuropathy, plasma protein, Mendelian randomization, therapeutic target, genome-wide association study

## Abstract

**Background:**

Peripheral neuropathy (PN) is a common neurological disorder, and circulating plasma proteins with causal genetic evidence are a major source of therapeutic targets. This study identifies several potential plasma proteins that are causally related to PN risk, providing new insights into protein-mediated pathogenesis of PN and potential targets for novel therapies.

**Methods:**

To identify potential therapeutic targets for PN, we employed two-sample Mendelian randomization (MR) to identify plasma proteins associated with six common PN. First, we screened for proteins related to PN using genome-wide association studies (GWAS), obtaining genetic data on plasma proteomes from 35,559 Icelanders. Summary data for six common PN, including Carpal Tunnel Syndrome (CTS), Trigeminal Neuralgia (TN), Alcoholic Neuropathy (AIP), Drug-induced Neuropathy (DIP), Diabetic Neuropathy (DP), and Guillain-Barré syndrome (GBS), were obtained from the FinnGen database. Two-sample MR and colocalization analyses were then conducted to further identify protein-PN pairs with presumed causal relationships. Enrichment analysis of positive proteins revealed potential biological processes and pathways. Based on drug-gene interaction analysis, we ultimately identified causal proteins associated with PN that could serve as potential drug targets for treating PN.

**Results:**

Through MR analysis, we identified eight proteins (UBC12, SEM4C, IL23R, Prothrombin, CBS, Microglobulin, MATN4, COLEC12) with causal relationships to PN. We found that UBC12 is a protective factor for DP and CTS, while the remaining proteins are risk factors. Further colocalization analysis showed a posterior probability of hypothesis 4 (PPH4) less than 0.75, indicating no positive colocalization results were found. From the pathway enrichment analysis, we discovered that the proteins were mainly concentrated in pathways related to defense response to bacterium, receptor signaling pathway via STAT, cell killing, negative regulation of cytokine production, and leukocyte mediated immunity. Finally, in Drug-Gene Interaction database (DGIdb), we identified three protein-coding genes (IL23R, F2, CBS) as potential drug targets for PN.

**Conclusion:**

Mendelian randomization studies confirm the causal relationship between genetically predicted PN-related risk and genetically predicted plasma protein abundance. Plasma proteins, as biomarkers associated with PN, can provide potential drug targets for etiological intervention research in PN.

## Introduction

Peripheral neuropathy (PN) is a common neurological disorder that encompasses diseases resulting from structural or functional damage to the peripheral nervous system due to various causes. The causes of peripheral neuropathy, ranked by incidence, include diabetes, impaired glucose tolerance, idiopathic, familial, vitamin deficiency or excess, thyroid disease, inflammatory or autoimmune conditions, paraneoplastic syndromes, drug-induced, and toxic or heavy metal exposure (1). The prevalence of peripheral neuropathy in the general population is approximately 1% (2), rising to as high as 50% in adults over 85 years old (3). Symptoms range from pain and frequent falls to amputations resulting from unnoticed injuries (4, 5). Currently, treatment options for PN are limited, mostly focusing on symptom management, such as neuroprotective therapies. Unfortunately, these medications cannot completely cure the disease and can only slow its progression to a certain extent. For example, one of the most common forms of PN is distal symmetric polyneuropathy, particularly in diabetic patients, who are at risk for ulcers and amputations (6). Given the high prevalence and disability risk of PN, further understanding of its pathogenesis is needed.

Plasma proteins play a key role in many biological processes, with circulating proteins always acting as primary regulatory factors in molecular pathways and being major sources of drug targets (7). Recently, thousands of protein Quantitative Trait Loci (pQTL) for plasma proteins have been identified through genome-wide association studies (GWAS) (8). These studies not only test the causal impact of plasma proteins on PN but also hold the potential to identify possible biomarkers and assess risk and protective factors associated with PN. Han et al. systematically identified causal relationships between 10 plasma proteins and Carpal Tunnel Syndrome from a biomolecular perspective using the largest GWAS summary statistics to date for two-sample MR analysis and mediation analysis (9). One study analyzed the correlation between circulating C1q/tumor necrosis factor-related protein 3 (ctrp3) concentrations in patients with diabetic peripheral neuropathy and several metabolic parameters, showing that ctrp3 concentrations were significantly reduced and positively correlated with nerve conduction velocity, suggesting that ctrp3 may serve as a predictive indicator of nerve conduction damage in patients with diabetic peripheral neuropathy (10). Another study conducted a randomized controlled trial to explore the relationship between the serotonin transporter gene (5-HTTLPR) and susceptibility to trigeminal neuralgia and pain severity (11). The etiology and classification of PN are numerous, and the causal relationship between plasma proteins and PN remains unclear. Establishing causal relationships can deepen the understanding of PN mechanisms and guide clinical interventions based on plasma protein profiles for PN. Using MR to integrate GWAS and pQTL data can help identify drug targets in advance, reduce experimental bias, and minimize confounding factors. This approach effectively utilizes experimental resources and time, avoids redundant work, and accelerates the research and development process. Therefore, clarifying the causal relationships between plasma proteins and various PNs is a matter of urgency.

Mendelian randomization (MR) is a statistical method that uses genetic variation (single nucleotide polymorphisms, SNP) as instrumental variables (IVs) to assess unexpected associations between exposure and outcomes (12). MR analysis relies on three assumptions: (1) the association assumption: SNPs are strongly correlated with the exposure factor; (2) the independence assumption: SNPs are independent of confounding factors; (3) the exclusivity assumption: SNPs can only affect the outcome through the exposure factor. By predicting protein levels using these IVs, we can evaluate the causal relationship between PN and proteins.

As the research on plasma proteins and PN from GWAS has gradually increased and is publicly available, this provides a certain research foundation for this study. The aim of this study is to identify causal proteins associated with the risk of PN across the proteome and explore new potential drug targets for PN.

## Materials and methods

### Study design

**Figure 1:**
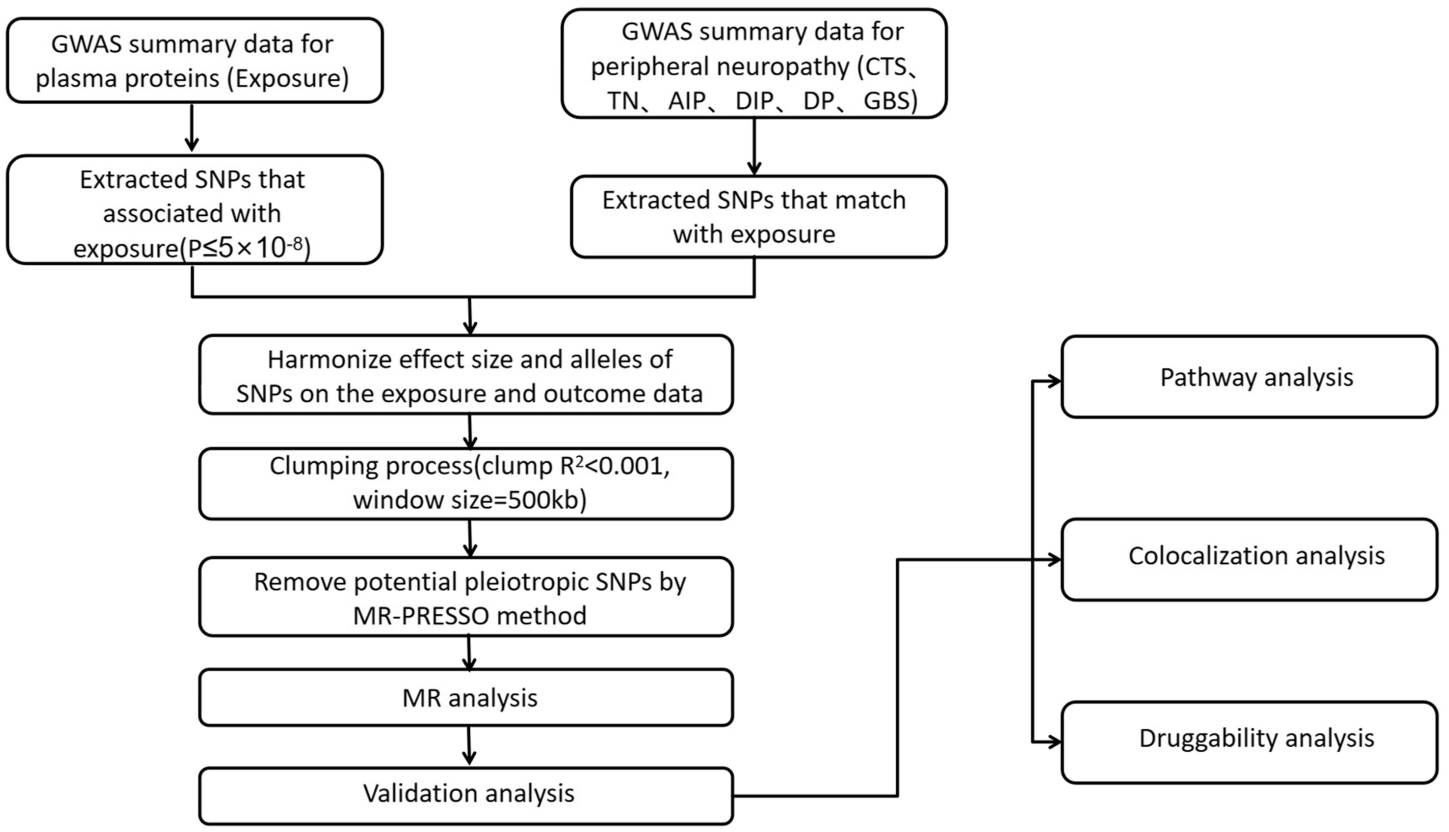
Instrumental variables selection and analysis process flow chart. MR, Mendelian randomization; CTS, carpal tunnel syndrome; TN, trigeminal neuralgia; AIP, alcohol induced polyneuropathy; DIP, drug-induced polyneuropathy; DP, diabetic polyneuropathy; GBS, guillain-barre syndrome.

The analysis of this study utilized publicly available GWAS summary statistics, which did not require ethical approval. Figure 1 shows the research process. Based on the dataset of quantitative trait loci for plasma proteins and summary statistics from six large-scale PN GWAS, we sequentially identified plasma proteins associated with PN through GWAS, MR analysis, and colocalization analysis, conducting a protein-centric protein group analysis to further screen potential drug targets for PN treatment.

### Data source

#### Genome-wide association studies summary statistics of plasma proteins

The GWAS summary statistics for the plasma proteome come from Decode Genetics,^1^ which includes 35,559 Icelanders (13) (Table 1). Plasma samples from all participants were measured using the Soma Scan version 4 assay (SomaLogic), resulting in 4,907 plasma protein levels.

**Table 1:** Data sources for studied phenotypes.

Study	Phenotype	Cases	Controls	PMID
deCODE	Plasma protein	35,559	–	34,857,953
FinnGen R9	Carpal Tunnel Syndrome	22,426	330,377	NA
FinnGen R9	Alcohol Induced Neuropathy	249	370,790	NA
FinnGen R9	Drug-induced Neuropathy	208	370,790	NA
FinnGen R9	Diabetic Neuropathy	1,048	374,434	NA
FinnGen R9	Guillain-barre syndrome	415	370,790	NA
FinnGen R9	Trigeminal Neurogia	1,599	330,377	NA

#### Genome-wide association studies summary statistics of PN

The GWAS summary statistics for PN were sourced from the FinnGen database^2^ (Table 1). These data include CTS (22,426 cases, 330,377 controls), TN (1,599 cases, 330,377 controls), AIP (249 cases, 370,790 controls), DIP (208 cases, 370,790 controls), DP (1,048 cases, 374,434 controls), and GBS (415 cases and 370,790 controls).

#### Instrumental variables selection

A series of quality control standards were employed to filter qualified genetic IVs. Specifically, (1) SNPs associated with the exposure were at genome-wide significance level (GWAS, *p* ≤ 5 × 10^−8^); (2) due to the complex linkage disequilibrium (LD) structure of SNPs within the human major histocompatibility complex (MHC) region, we excluded SNPs located in the MHC region (GRCh38: chr6: 29 to 33 Mb; GRCh37: chr6: from 26 to 34 Mb) (14); (3) SNPs for each protein were clustered, retaining only independent SNPs. The clustering LD threshold was set at r^2^ = 0.001, with a clustering window size of 500 kb; (4) SNPs associated with fewer than 5 proteins were retained, while SNPs associated with 5 or more proteins were considered highly pleiotropic.

The strength of IVs was verified by calculating *F*-statistics using the formula.


$$\frac{R^{2}\times \left(N-1-k\right)}{\left(1-R^{2}\right)\times k}$$

where *R*^2^ is the proportion of variance explained by the IVs, N is the sample size, and k is the number of IVs. (5) IVs of the *F*-statistic<10 were considered as weak IVs and excluded in the subsequent analysis. The remaining SNPs were used in the subsequent MR analysis (15).

### Statistical analysis

We further conducted a two-sample MR analysis and performed a series of sensitivity analyses to assess the potential causal relationship of proteins with PN. The MR analysis adhered to the STROBE-MR Statement (16) (Supplementary Table S3), primarily involving the selection of IVs, evaluation of IVs, MR analysis, and sensitivity analysis. For the MR analysis, we used the Wald Ratio method for proteins with only one IV, the fixed-effect inverse variance weighted (IVW) method (17) for proteins with two or three IVs, and the random-effects IVW method for proteins with four or more IVs. Notably, the random-effects model, by allowing for overdispersion in the regression model, can account for heterogeneity among IVs. It has been reported that all causal estimates of plasma proteins for PN are odds ratios, indicating changes in PN risk with each SD change in protein abundance. To further strengthen the validity of the MR results, we conducted MR-Egger and weighted median MR analyses. In short, MR-Egger can detect and correct for horizontal pleiotropy, where the intercept can be used to identify the presence of horizontal pleiotropy (18). The weighted median method can provide consistent estimates even when up to 50% of the information comes from invalid IVs and allows for a degree of heterogeneity among IVs (19). The Cochran’s Q test was used to assess the heterogeneity of individual causal effects. The MR-Egger intercept term was also used to evaluate horizontal pleiotropy. When the *p*-values from these tests are less than 0.05, it typically indicates the presence of heterogeneity or pleiotropy. The final MR results were determined by a combination of MR-Egger results and weighted median estimates. Finally, we used FDR (Benjamini-Hochberg method) for multiple corrections in the MR analysis; when conducting multiple comparisons, we adopted a *p*-value threshold of <1.8 × 10^−6^ (0.05/27,498) as the significant *p* value threshold. This strict threshold was chosen to ensure the robustness and reliability of the validation results, thereby further enhancing the credibility of the findings. We utilized R software (4.3.2) with packages such as TwoSampleMR, coloc, and MendelianRandomization for the MR analysis.

### Sensitivity analysis

For sensitivity analysis, we first fitted the MR-Egger model (20), followed by further sensitivity analysis through MR-Egger regression analysis and leave-one-out analysis, considering a significant intercept term (*p* < 0.05) as an indicator of horizontal pleiotropy. Then, we calculated Cochran’s Q statistic to assess the heterogeneity of proteins with multiple IVs. Finally, leave-one-out analysis was conducted by re-estimating the MR association after sequentially removing each variant.

### Colocalization analysis

To detect whether the identified proteins and PN share common causal variants in a given region, we performed colocalization analysis using the coloc.abf function. We set the window size to ±500 kb, centering on the IV for each protein-PN pair. We used default priors, with p_1_ as 1 × 10^−4^, p_2_ as 1 × 10^−4^, and p_12_ as 1 × 10^−5^. The evidence for colocalization was assessed using the posterior probability (PP) of hypothesis 4 (which indicates that both the protein and disease are associated and driven by the same causal variant). We used PP.H4 > 0.75 as the threshold, indicating that the association is highly colocalized (21).

### Enrichment analysis and PPI network

To explore the possible biological mechanisms of PN-related proteins identified by MR, we conducted enrichment analysis on proteins with positive results and filtered the results to include only pathways with a corrected *p* < 0.05 for significance. We performed enrichment analysis using both the metsape and Kyoto Encyclopedia of Genes and Genomes (KEGG) databases. Metscape calculates pairwise similarity between any two enriched terms based on Kappa test scores and automatically clusters the enriched terms into non-redundant groups. *p* values were generated using hypergeometric tests and corrected using the Benjamini-Hochberg FDR method (22). The parameters for “Min Overlap,” “*P* Value Cutoff,” and “Min Enrichment” were set to default values. Additionally, we used the STRING database to infer enriched protein clusters and generate PPI networks to explore the interactions between important proteins identified in the MR analysis (23).

### Druggable targets exploration

To explore whether the identified proteins could serve as targets for existing drugs or druggable gene targets, we examined the interactions between these proteins (or genes) and drugs using the Drug-Gene Interaction Database (DGIdb).^3^ DGIdb provides search and filtering for drug-gene interactions and pharmacogenomics information. This database integrates information from DrugBank, PharmGKB, ChEMBL, Drug Target Commons, and the Therapeutic Target Database (TTD), as well as over 30 other reliable sources, encompassing more than 40,000 genes and 10,000 drugs. It involves over 100,000 drug-gene interactions or belongs to one of 42 potential drug-gene categories and has been widely used to prioritize potential drug targets for diseases (24).

## Results

### Causal effects of plasma proteins on PN in the discovery stage

**Figure 2:**
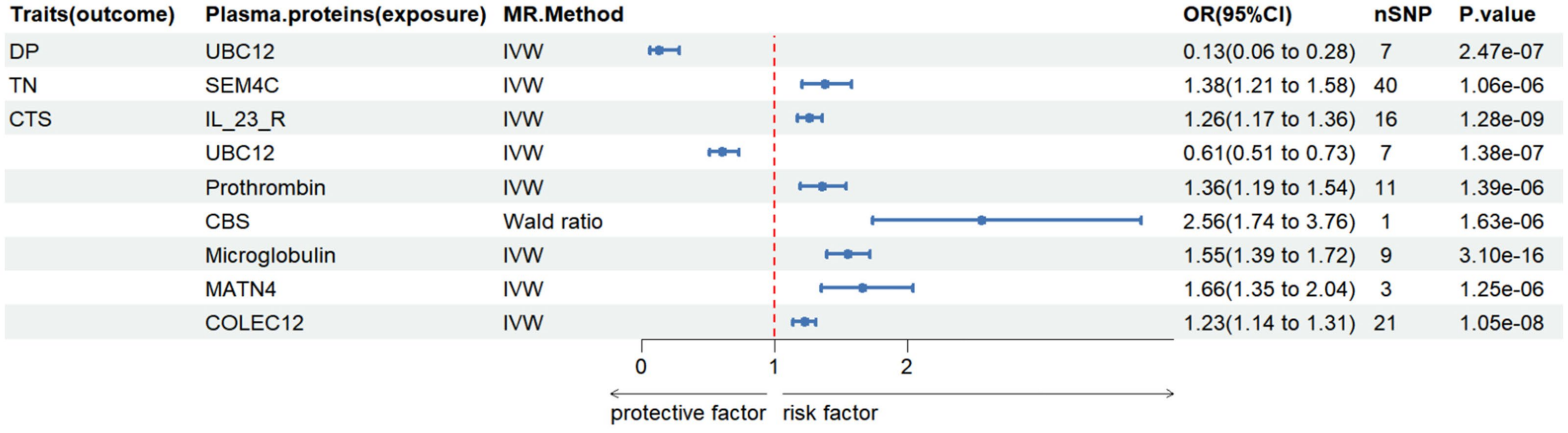
The causal relationship between plasma proteins and Diabetic polyneuropathy in discovery samples. IVW, inverse variance weighted, nSNP, number of instrumental variables, OR, odd ratio, CI, confidence interval.

In this study, we conducted MR analysis of two samples to investigate the causal relationships between plasma proteins and six common PN (CTS, TN, AIP, DIP, DP, and GBS). We identified 9 meaningful protein-PN pairs, including 1 pair for DP, 1 pair for TN, and 7 pairs for CTS. The IV *F*-statistics ranged from 40.259 to 215.533, all greater than 10, indicating a low likelihood of weak IV bias. Table 2 shows the significant MR analysis results in the discovery sample. However, there was no causal relationship between AIP and DIP, and GBS had a protein level pleiotropy test less than 0.05, thus it was excluded. As shown in Figure 2, elevated levels of 7 proteins (SEM4C, IL_23_R, prothrombin, CBS, Microglobulin, MATN4, COLEC12) were associated with an increased risk of PN (odds ratio (OR) range from 0.61 to 2.56). In contrast, elevated levels of one protein (UBC12) were associated with a decreased risk of PN (OR range from 0.13 to 0.61).

**Table 2:** Significant MR analysis results in the discovery samples.

Traits (outcome)	Plasma protein (exposure)	MR methods	NO.SNP	*F*-statistics	OR	95% CI	*p*-value
DP	UBC12	IVW(fixed)	7	40.259	0.132	0.061–0.284	2.47 × 10^−7^
TN	SEM4C	IVW(fixed)	40	215.534	1.384	1.215–1.578	1.06 × 10^−6^
CTS	IL_23_R	IVW(fixed)	16	100.249	1.261	1.170–1.359	1.28 × 10^−9^
	UBC12	IVW(fixed)	7	40.259	0.609	0.507–0.733	1.38 × 10^−7^
	Prothrombin	IVW(fixed)	11	58.411	1.357	1.199–1.536	1.39 × 10^−6^
	CBS	Wald ratio	1	41.292	2.561	1.744–3.762	1.63 × 10^−6^
	Microglobulin	IVW(fixed)	9	76.762	1.545	1.392–1.715	3.10 × 10^−16^
	MATN4	IVW(fixed)	3	42.097	1.662	1.353–2.041	1.25 × 10^−6^
	COLEC12	IVW(fixed)	21	74.647	1.226	1.143–1.315	1.05 × 10^−8^

No. SNP is the number of SNPs being used as IVs. MR, Mendelian randomization; SNP, single-nucleotide polymorphisms; IVW, inverse-variance weighted; OR, odds ratio; CI, confidence interval; DP, Diabetic Polyneuropathy; GBS, Guillain-barre Syndrome; TN, Trigeminal Neuralgia; CTS, Carpal Tunnel Syndrome.

### Pathway analysis

**Figure 3:**
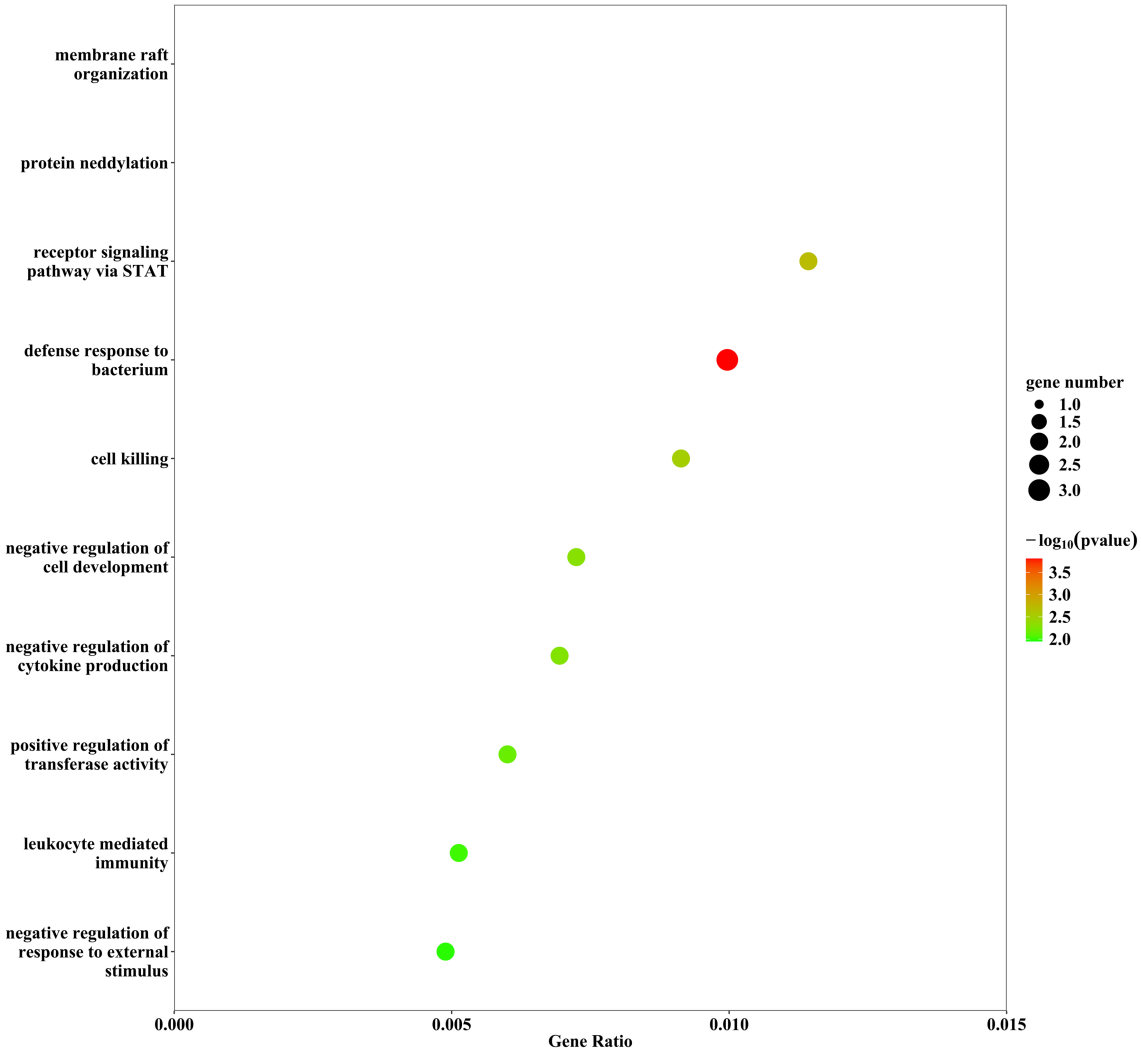
Bubble chart for KEGG enrichment analysis.

**Figure 4:**
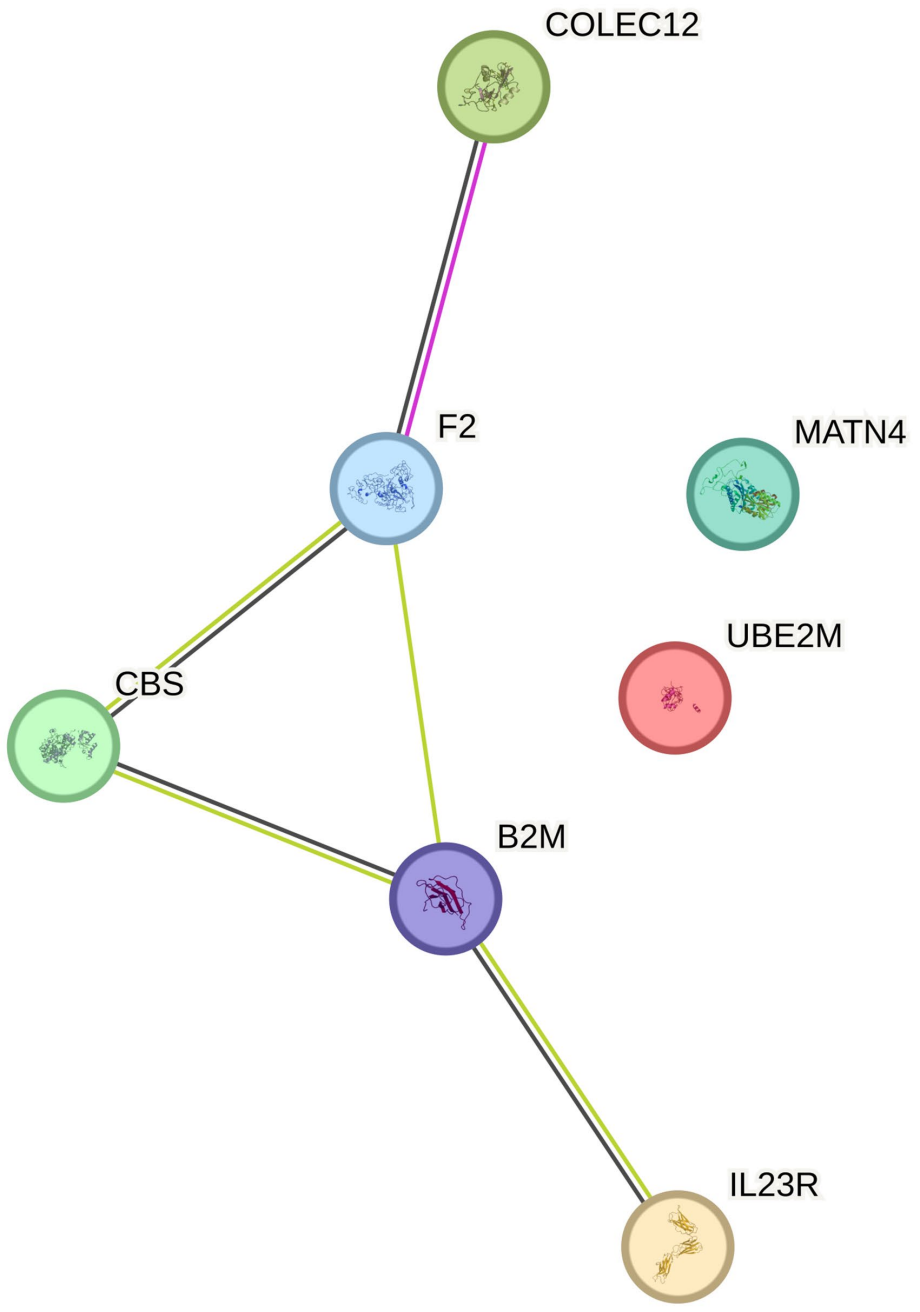
PPI (protein–protein interaction network) for positive protein analysis sensitivity analysis and colocalization analysis.

We further conducted enrichment analysis on 8 proteins, and for KEGG enrichment analysis, we found a total of 10 pathways associated with PN (Figure 3), We discovered that these proteins primarily participate in defense response to bacterium, receptor signaling pathway via STAT, cell killing, negative regulation of cytokine production, and leukocyte mediated immunity. Subsequently, the PPI network diagram further illustrated the detailed interactions between the important proteins identified in the MR analysis (Figure 4).

In our preliminary analysis, we fitted the MR-Egger model and considered significant intercept terms (*p* < 0.05) as indicators of horizontal pleiotropy. We found no significant heterogeneity and pleiotropy among the proteins in the MR analysis. The resulting 8 proteins underwent further sensitivity analysis, and the results were robust [(1) The direction of MR estimates in sensitivity analysis for each case was consistent with the primary analysis; (2) No imbalanced horizontal pleiotropy was observed] (Supplementary Table S1), indicating that causal effect estimates were not influenced by directional pleiotropy. Leave-one sensitivity analysis indicated that all identified causal relationships were not driven by any single SNP (Supplementary Figure). Therefore, all selected protein level gene IVs should be considered valid for MR analysis. Further positive proteins underwent colocalization analysis using coloc software, but PPH4 was less than 0.75, indicating that the target proteins did not have positive colocalization results.

### Drug target identification

Since most drugs exert their therapeutic effects by targeting proteins, we ultimately explored whether the eight proteins identified through comprehensive analysis could serve as potential therapeutic targets. In the DGIdb database, we identified three protein-coding genes (IL23R, F2, CBS) as potential drug treatment targets (Table 3) through drug-gene interactions, for further mechanistic studies and drug development.

**Table 3:** The result druggability of causal proteins.

Proteins	Protein (full name)	Entrez Gene Symbol	UniProt ACC	Uniprot ID	Drug	Regulatory approval	Indication	Interaction score
IL_23_R	Interleukin-23 receptor	IL23R	Q5VWK5	IL23R_HUMAN	AZATHIOPRINE	Approved	–	0.921
					CELECOXIB	Approved	NSAID	0.522
					INFLIXIMAB-DYYB	Approved	DMARD, anti-inflammatory agent	1.129
Prothrombin	Prothrombin	F2	P00734	THRB_HUMAN	DABIGATRAN	Approved	Anticoagulant	0.843
					MELAGATRAN	Not approved	–	0.2808574
					DABIGATRAN ETEXILATE MESYLATE	Approved	–	1.685
					PYRIDOXAL PHOSPHATE ANHYDROUS	Not approved	–	0.843
					CHEMBL:CHEMBL1083499	Not approved	–	1.685
					ATECEGATRAN METOXIL	Not approved	Anticoagulant	3.370
					ICHORCUMAB	Not approved	–	1.685
					LUSUTROMBOPAG	Approved	–	0.562
					APRAMIDE A	Not approved	–	3.370
					BORTEZOMIB	Approved	Antineoplastic agent	0.039
					AVATROMBOPAG	Approved	–	0.481
					CYANIN	Not approved	–	1.685
					AZD-8165	Not approved	–	1.685
					ODIPARCIL	Not approved	–	1.685
					ARGATROBAN	Approved	Anticoagulant	2.528
					ANISINDIONE	Approved	Anticoagulants	0.337
					DESIRUDIN	Approved	Antithrombotic	2.247
					CYANIDIN	Not approved	–	1.685
					CATECHIN	Approved	–	0.129
					ENOXAPARIN SODIUM	Approved	Anticoagulant	0.562
					EPICATECHIN	Not approved	–	0.140
					TAMOXIFEN	Approved	Hormonal, Antineoplastic Agents	0.055
					HIRUDIN	Not approved	–	1.685
					ANTITHROMBIN ALFA	Approved	Antithrombotic	0.374
					FLOVAGATRAN	Not approved	Anticoagulant	1.685
					CHEMBL:CHEMBL586628	Not approved	–	1.685
					DPOC-4088	Not approved	–	1.685
					QUERCETIN	Approved	–	0.021
					PEGMUSIRUDIN	Not approved	–	1.685
					BIVALIRUDIN	Approved	Antiplatelet agent	6.741
					VITAMIN K3	Approved	For reducing EGFR-inhibitor-induced dermatological side effects	0.058
					SILIBININ	Not approved	–	0.140
					SR-123781A	Not approved	–	0.843
					XIMELAGATRAN	Approved	Anticoagulants	5.055
					TINZAPARIN SODIUM	Approved	For treatment of cystic fibrosis, for treatment of pelvic pain of bladder origin and interstitial cystitis, antithrombotic, anticoagulant, Anticoagulants	0.099
CBS	Cystathionine beta-synthase	CBS	P35520	CBS_HUMAN	VITAMIN B6	Approved	–	3.501
					METHIONINE	Approved	–	4.201
					3H.2-DEOXYGLUCOSE	Not approved	–	2.625
					COMPOUND 30	Not approved	–	10.501
					BETAINE	Approved	–	2.100

## Discussion

In our study, we conducted MR analysis on two samples to investigate the causal relationship between plasma proteins and six common peripheral neuropathies (CTS, TN, AIP, DIP, DP, GBS). Through MR analysis, we identified eight proteins (UBC12, SEM4C, IL_23_R, prothrombin, CBS, Microglobulin, MATN4, and COLEC12) with causal relationships to peripheral neuropathy. UBC12 was associated with DP, SEM4C with TN, and seven proteins (IL23R, UBC12, prothrombin, CBS, Microglobulin, MATN4, COLEC12) were associated with CTS. Further co-localization analysis indicated that no shared genetic variations were found for PPH4.

Enrichment analysis identified signaling pathways associated with peripheral neuropathy, and further PPI protein networks deepened our understanding of the mechanisms related to these proteins and their interrelations. The signaling pathways primarily included defense response to bacterium, receptor signaling pathway via STAT, cell killing, negative regulation of cytokine production, and leukocyte mediated immunity, with IL23R mainly enriched in pathways related to defense response to bacterium, receptor signaling pathway via STAT, cell killing, and leukocyte mediated immunity. These results suggest that peripheral nerve injury is largely mediated by inflammation and cellular immunity. In an animal study of painful diabetic neuropathy (25), it was found that there was significantly increased glial proliferation and upregulation of phosphorylated signaling kinases (including pERK, pAKT, and pSTAT 3) in the spinal cords of db/db mice. The non-competitive NMDA receptor antagonist MK-801 could alleviate mechanical allodynia and the upregulation of pERK, pAKT, pSTAT 3, as well as the production of TNF-α and IL-6.

We further conducted drug-gene interaction studies, and among the eight positive proteins, we found that only three proteins (IL_23_R, prothrombin, CBS) had corresponding drug-gene interactions and explored potential drug targets. These three proteins were positively correlated with the risk of peripheral neuropathy, while protective protein UBC12 and other related proteins’ targeted drugs are still in the development stage. UBC12 is a common protective factor for DP and CTS; for example, the function of UBC12 is to accept the ubiquitin-like protein NEDD8 from the UBA3-NAE1 E1 complex and catalyze its covalent linkage to other proteins. In studies of diabetic peripheral neuropathy (DPN), sumoylation involves small ubiquitin-like modifications and is an important neuroprotective mechanism for type 2 diabetic sensory neurons. Its absence leads to oxidative stress and damage to the respiratory chain, resulting in energy depletion and subsequent sensory neuron loss (26). Therefore, in future laboratory studies, we can look for specific drug targets for treating peripheral neuropathy in proteins similar to UBC12 and other undeveloped proteins.

From the analysis of enriched pathways, we can find that IL23R is enriched in most signaling pathways (Supplementary Table S2). Focusing on drug-gene interactions, we found that the approved clinical therapeutic drugs for IL23R include Azathioprine, Celecoxib, and INFLIXIMAB-DYYB. IL23R is an interleukin-23 receptor formed by the binding of IL-12rb1, which binds to IL-23 and may mediate the stimulation of T cells, NK cells, and some macrophages/myeloid cells by activating the Jak–STAT signaling cascade. The positive protein IL-23 requires macrophages to release IL-17A to induce mechanical pain in female mice, revealing that the IL-23/IL-17A/TRPV1 axis regulates female-specific mechanical pain through neuroimmune interactions (27). The results of this study and laboratory research indicate that the pathogenesis of peripheral neuropathy is associated with factors such as mitochondrial dysfunction, neurotrophic factor degeneration and proliferation disorders, and neuroimmune regulatory processes. Other studies have shown that clinically approved therapeutic drugs such as Azathioprine, Thalidomide, Pentoxifylline, and Vitamin C can be used to treat neurological diseases associated with human T-cell lymphotropic virus type 1 (HTLV-1) (28).

Secondly, from the KEGG analysis, we also found that Prothrombin is enriched in the vast majority of pathways (Supplementary Table S2). Thrombin cleaves bonds after arginine and lysine, converting fibrinogen into fibrin, activating factors V, VII, VIII, XIII, and forming a complex with the coagulation regulatory protein protein C, playing a role in blood balance, inflammation, and wound healing (29). Thrombin can also trigger the production of pro-inflammatory cytokines such as MCP-1/CCL 2 and IL 8/CXCL 8 in endothelial cells (30). In an animal experiment, the authors studied the balance between thrombin and its serine protease inhibitor protein I (PNI) after sciatic nerve injury in mice. The data indicated that nerve injury first induced the synthesis of prothrombin, which was subsequently converted into active thrombin. The thrombin induced by nerve compression was followed by the production of functionally active PNI, which may be its direct inducing cause (31).

At the same time, we also discovered a new protein (SEM 4C) that has a potential causal effect on peripheral neuropathy (PN). SEM 4C is a cell surface receptor for PLXNB2, playing an important role in cell–cell signaling and is an essential protein required for normal brain development, axon guidance, and cell migration. It may also act as a signaling receptor that plays a role in myogenic differentiation by activating stress-activated MAPK cascades (32). SEM 4C and its receptor Plexin B are expressed in sensory neurons and are associated with pain in mouse models of inflammatory pain (33). Currently, targeted drugs for SEM 4C are still in the development stage, and further experimental research is needed to explore the mechanisms and targeted therapies. The above results indicate that the druggable proteins identified in this study have the potential to become effective specific target proteins for PN, facilitating the development of PN drugs.

Our study has several limitations. First, since protein Quantitative Trait Loci (pQTL) GWAS is still in its early stages, the selection of instrumental variables (IV) is very limited. Second, the GWAS data used in this study is entirely derived from European populations, which may limit the generalizability of our findings to other ethnic groups. Third, although we used six currently available GWAS datasets related to PN, the sample size is still relatively small, which may increase the risk of bias and limit the statistical power of the study. Fourth, to better understand the therapeutic effects of the targeted proteins in our study, we need to design more targeted clinical trials to further elucidate the biological mechanisms behind the *in vivo* and *in vitro* experimental results.

In summary, our study identified six PN-related biomarkers and provided deeper insights into their pathogenesis. Through MR analysis, we identified nine plasma proteins associated with PN and screened three proteins (IL_23_R, prothrombin, CBS) as potential existing drug target proteins. There are also many target proteins (UBC12, SEM4C, Microglobulin, MATN4, COLEC12) for which drugs have not yet been developed, and these findings provide guidance and new directions for targeted therapy. Most importantly, rigorous experimental and clinical studies must be conducted to fully assess the practicality and effectiveness of these potential candidate drugs and to validate the current research findings.

## Data Availability

The original contributions presented in the study are included in the article/Supplementary material, further inquiries can be directed to the corresponding author.

## Footnotes

^1^ https://www.decode.com/summarydata/

^2^ https://www.finngen.fi/en/access_results

^3^ https://www.dgidb.org/
