# Supplementary figures and images for: Exploring causal correlations between plasma proteins and peripheral neuropathy: a Mendelian randomization
Source: Front Neurol. 2024 Aug 29;15:1431669. doi: 10.3389/fneur.2024.1431669 (PMC11390399; doi:10.3389/fneur.2024.1431669)

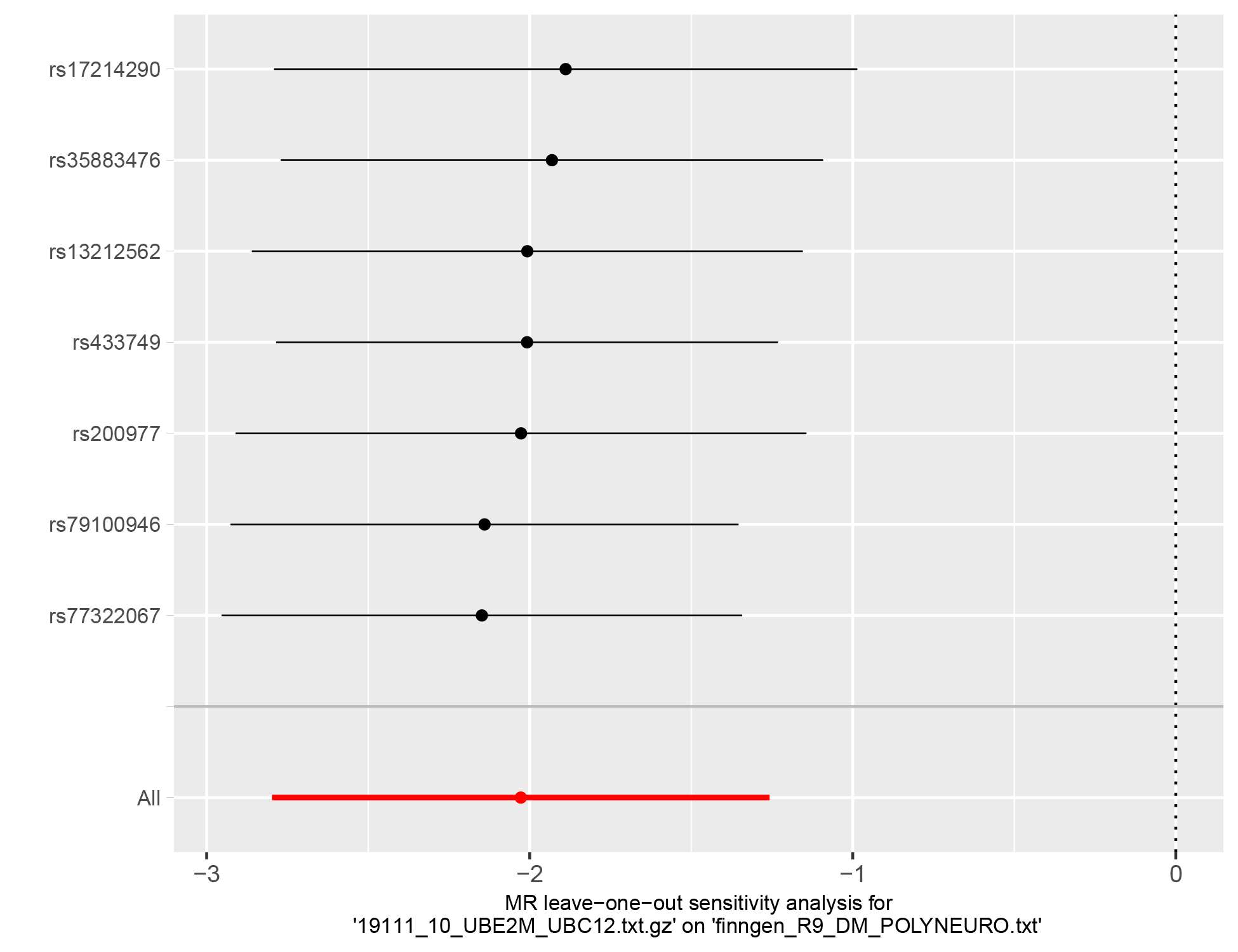


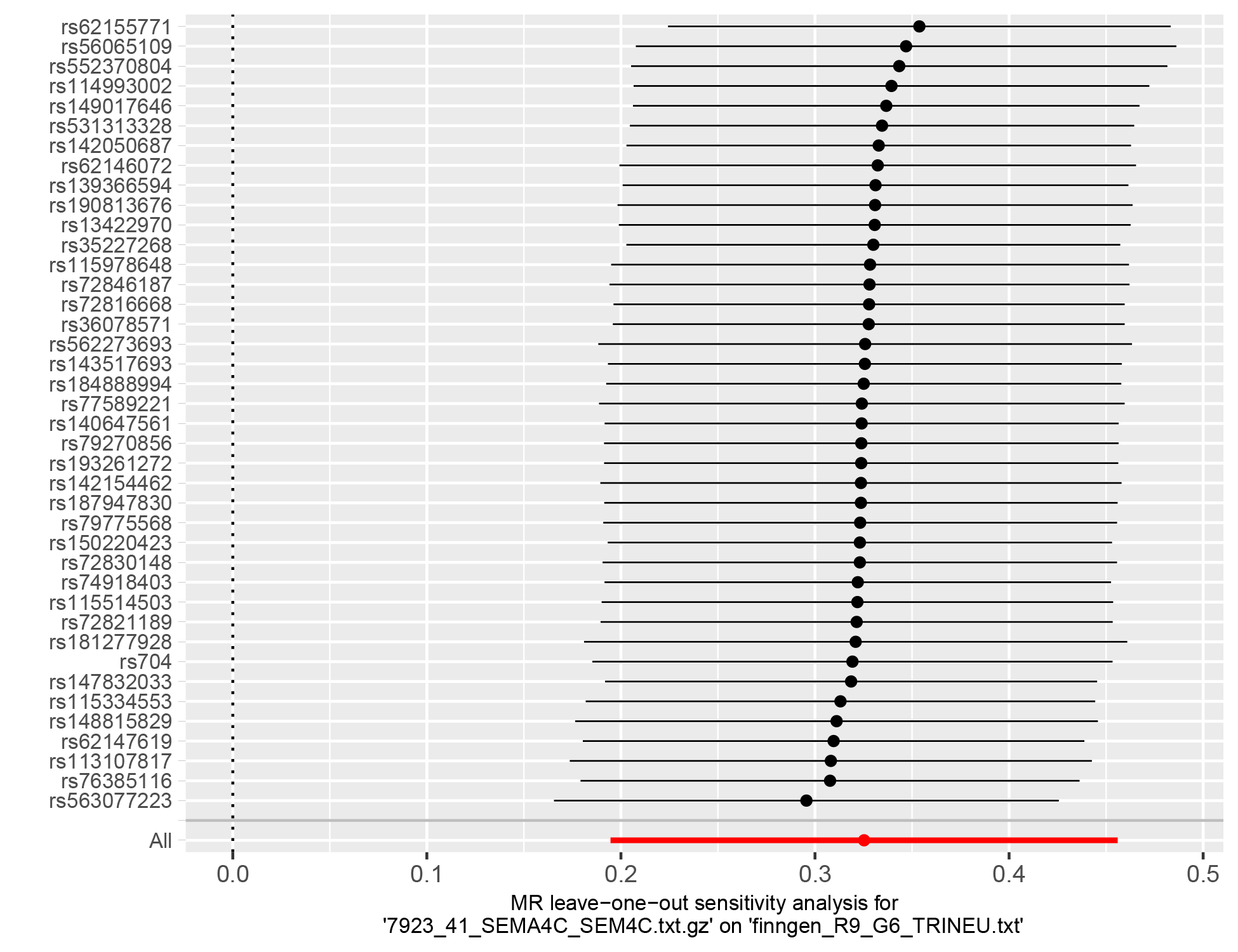


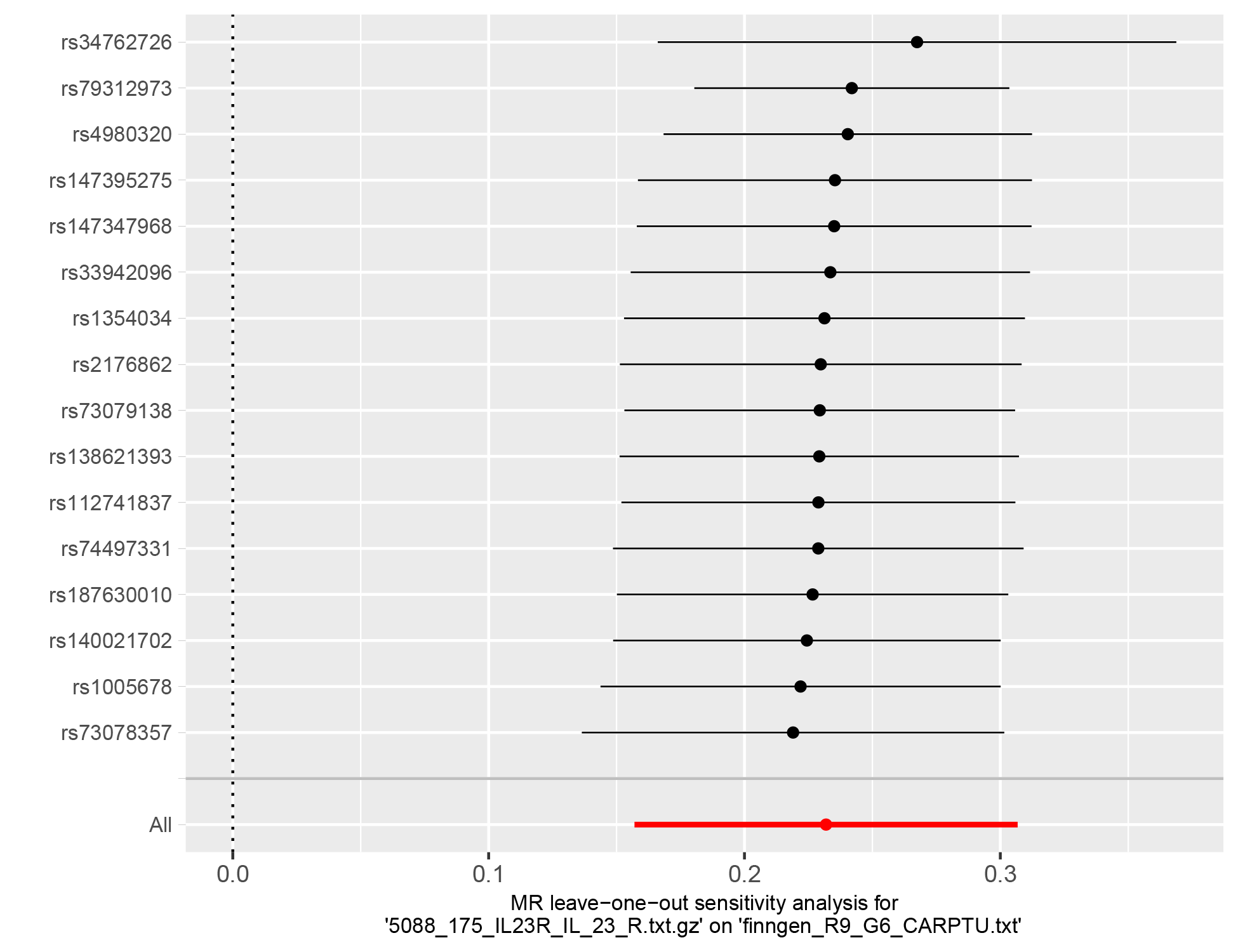


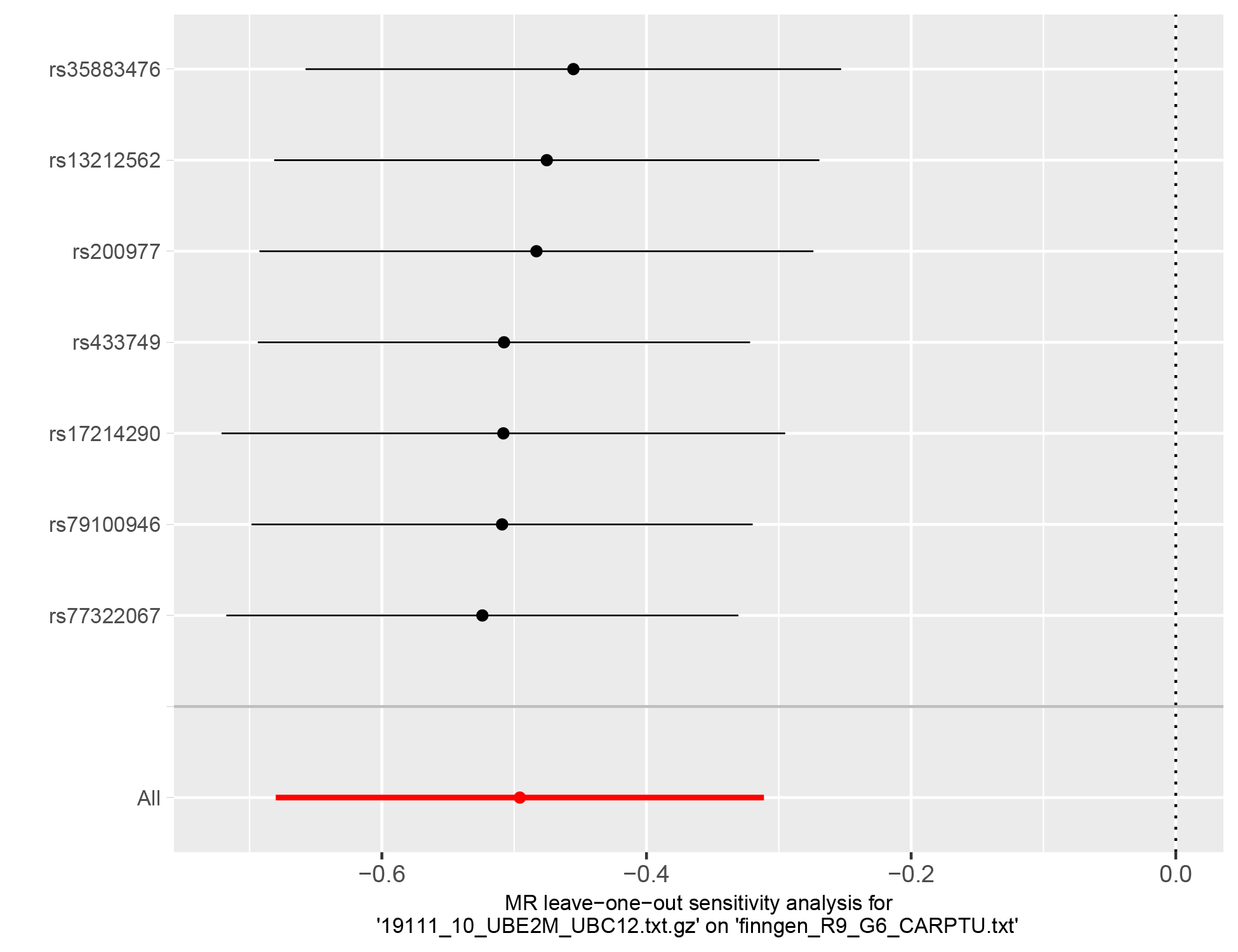


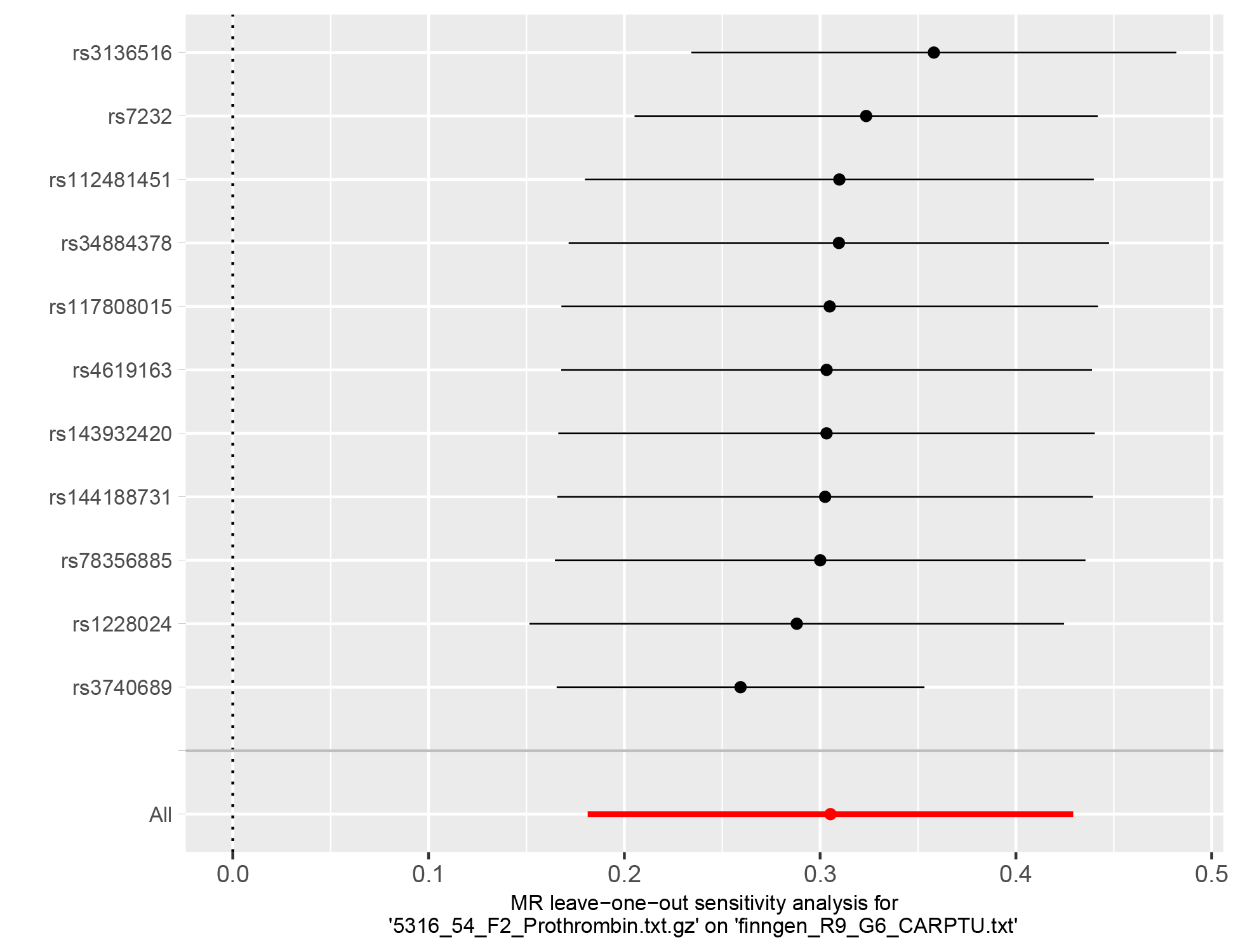

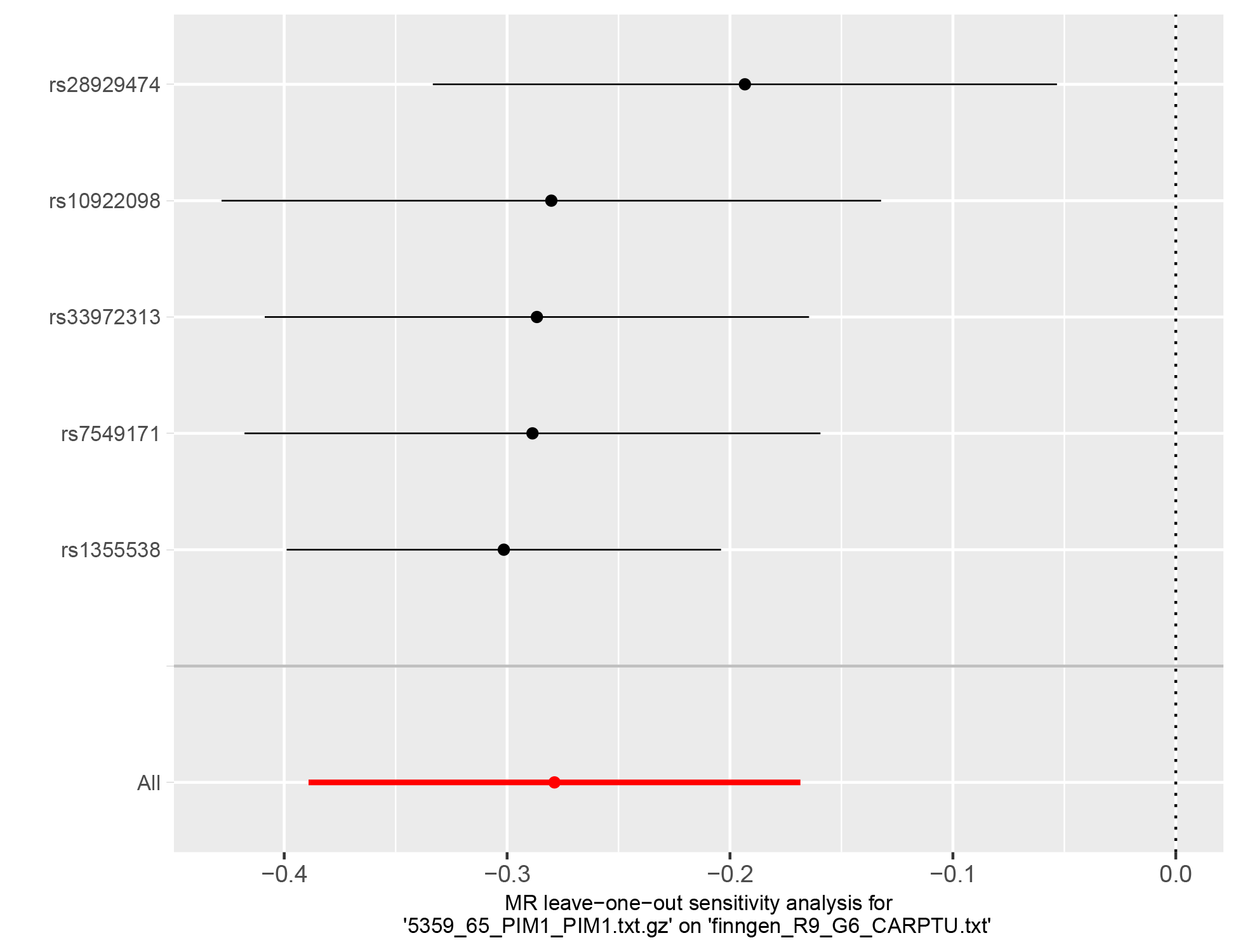


**Fig.S1**  **Leave-one-out plot for sensitivity analysis of causal protein with PN.**

Supplement: Supplementary file 1 [file Table_1.DOCX]
